# Chromosome 18q-Syndrome and 1p terminal duplication in a patient with bilateral vesico-ureteral reflux: case report and literature revision

**DOI:** 10.1186/1824-7288-39-6

**Published:** 2013-01-23

**Authors:** Elisa Brandigi, Francesco Molinaro, Anna Lavinia Bulotta, Rossella Angotti, Maria Pavone, Mario Messina

**Affiliations:** 1 Division of pediatric surgery, department of pediatrics, obstetrics and reproductive medicine, University of Siena, Siena, Italy

**Keywords:** Vesico-ureteral reflux, Deletion of 18q, Duplication of 1p

## Abstract

**Background:**

Vesico-ureteral reflux (VUR) is a dynamic event in which a retrograde flow of urine is present into the upper tracts. VUR may occur isolated or in association with other congenital abnormalities or as part of syndromic entities. We present a patient with a bilateral primary VUR, syndromic disease caused by a large deletion of 18q (18q21.3-qter) and terminal duplication of 1p (1p36.32-p36.33).

**Case report:**

The patient was 8 years old female with a disease including moderate growth retardation, psychomotor retardation, facial dysmorphism, single umbilical artery, umbilical hernia, urachal remnant, bilateral congenital clubfeet and renal-urinary disease. Chromosomal analysis and Array-CGH revealed two heterozygous chromosomal rearrangements: 1p terminal duplication and de novo 18q terminal deletion. She referred to our clinic to evaluation of bilateral hydronephrosis and right renal cortex thinning. Voiding cystourethrography demonstrated bilateral grade IV VUR and dimercaptosuccinic acid renal scintigraphy confirmed right renal cortex thinning and showed a cortical uptake of 75% of the left kidney and 25% of the right kidney. The patient underwent ureterovesical reimplantation after failure of 3 endoscopic submeatal Deflux injections with VUR resolution.

**Conclusions:**

This is the first report involving a patient with 18q-syndrome and contemporary presence of 1p chromosomal terminal duplication. The coexistence of two chromosomal rearrangements complicates the clinical picture and creates a chimeric disorder (marked by characteristics of both chromosomal anomalies). Kidney problems, primarily VUR is reported in 15% of patients affected by 18-q syndrome and no cases is reported in the literature regarding a correlation between VUR and 1p36 chromosomal duplication.

## Introduction

Vesico-ureteral reflux (VUR) is the most common disease of the urinary tract in children and affecting approximately 1% of all children [1].

The primary reflux may occur isolated or in association with other congenital abnormalities of kidney/urinary tract or as part of syndromic entities [2].

Syndromic VUR has an inherited Mendelian trasmission [3]. Several congenital syndromes associated with VUR as Renal coloboma o Brachio-otorenal syndrome were know [2]. The 18q syndrome affecting the patient that we present isn’t included among these syndromes despite some cases presenting VUR have been reported [4].

The 18q-syndrome is chromosomal disorder resulting from a total or partial deletion of the long arm of chromosome 18 [5]. The syndrome constitutes one of the most frequent autosomal syndrome in man and the clinical phenotype is variable because the breakpoint varies greatly and it is generally characterized by mental retardation, development delay, short stature and a specific pattern of cranofacial dysmorphisms, hearing loss, neurologic abnormalities such hypoplasia, hands and feet anomalies [5,6].

Chromosome 1p duplication is a very rare rearrangement and the most consistent manifestations are low birth weight, grow retardation, craniosynostosis, facial anomalies (flat nasal bridge, hiperthelorisme, cleft palade), hand malformation, congenital heart diseases [7].

We present a patient with a bilateral primary VUR, syndromic disease caused by a large deletion of 18q (18q21.3-qter) and terminal duplication of 1p (1p36.32-p36.33).

To our knowledge no patient has so far been reported to be affected by both 18q-syndrome and 1p36 duplication.

## Clinical report

The patient was a 8 years old female and she was the second child of a healthy and non consanguineous couple. The first-born is healthy. The patient was born at 40 weeks gestation by a normal spontaneous vaginal delivery. Her birth weight was 2700 gr, birth length was 48 cm and her head circumference was 31 cm (< III° percentile). She was noted at birth to have a single umbilical artery, umbilical hernia, renal-urinary disease and bilateral congenital clubfeet treated by an application of moulded plaster casts.

**Figure 1:**
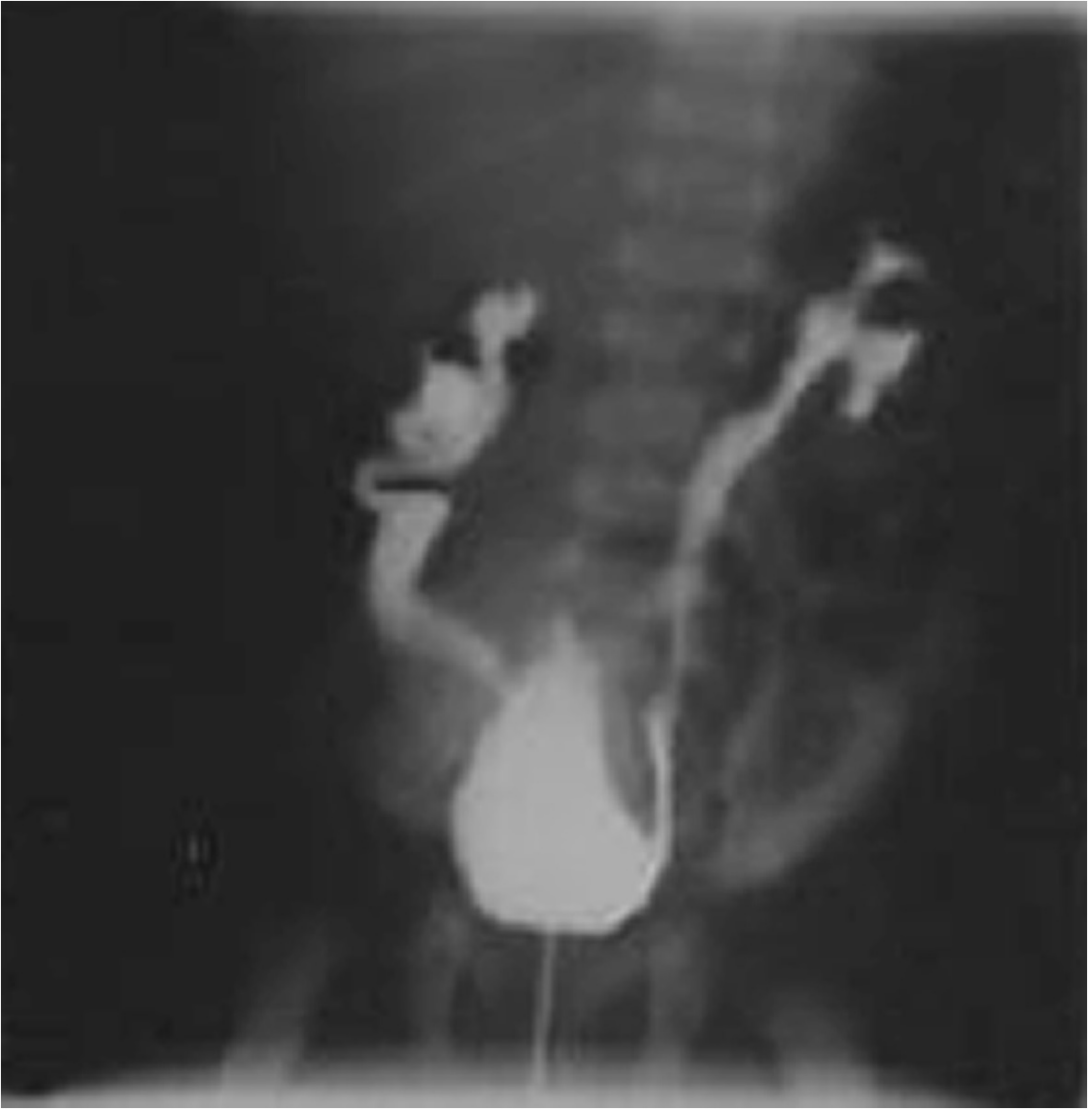
CUM imaging: IV° grade bilateral VUR.

At the birth a renal ultrasonography was performed to evaluate an hydronephrosis discovered during a prenatal ultrasonography. The renal ultrasonography showed a bilateral hydronephrosis and right renal cortex thinning. Voiding cystourethrography (VCUG) demonstrated bilateral grade IV VUR and urachal remnant later treated by a surgical approach (Figure 1).

A renal dimercaptosuccinic acid (DMSA) scan performed at 6 months confirmed right renal cortex thinning and revealed a cortical uptake of 75% of the left kidney and 25% of the right kidney.

The patient was initially treated with endoscopic submeatal bilateral injection of 0.5 cc Deflux at 30 months.

After this procedure, antibiotic prophylaxis (AP) with Cefixime (10 mg/Kg/dose) was administered until six month. During this period the patient didn’t show UTI signs but her growth was retarded. When she was two and a half years old an UTI occurred with high fever and positive of urine culture. A VCUG confirmed persistent of left grade III VUR so, she was undertaken to a subsequent left injection of 0,6 cc of Deflux®. Treatment with AP was continued and the result of urine culture performed every two months was negative. One year after this procedure a new VCUG was performed and the result is the same of the previous examination. For the persistent of a postoperative reflux she was undertaken at the age of five years old to a subsequent Deflux® injection with a failure of the treatment. The patient at 7 years of age was underwent with bilateral Glenn-Anderson’s ureterovesical reimplantation with VUR resolution.

Standard karyotype was performed on peripheral blood lymphocytes from the patient and both parents with GAG, QFQ banding techniques.

The Karyotype designation was 46,XX,del(18) (q21.3->qter) occurred de novo, as both parents had normal karyotype. Subsequently Array-CGH was done with Agilent Human Genome CGH Microarray Kit 44B) and confirmed a del(18) (q21.3->qter) involving 53 genes in the one chromosome 18, but furthermore revealed the presence of a terminal duplication of 1p (1p36.32-p36.33) involving 74 genes.

**Figure 2:**
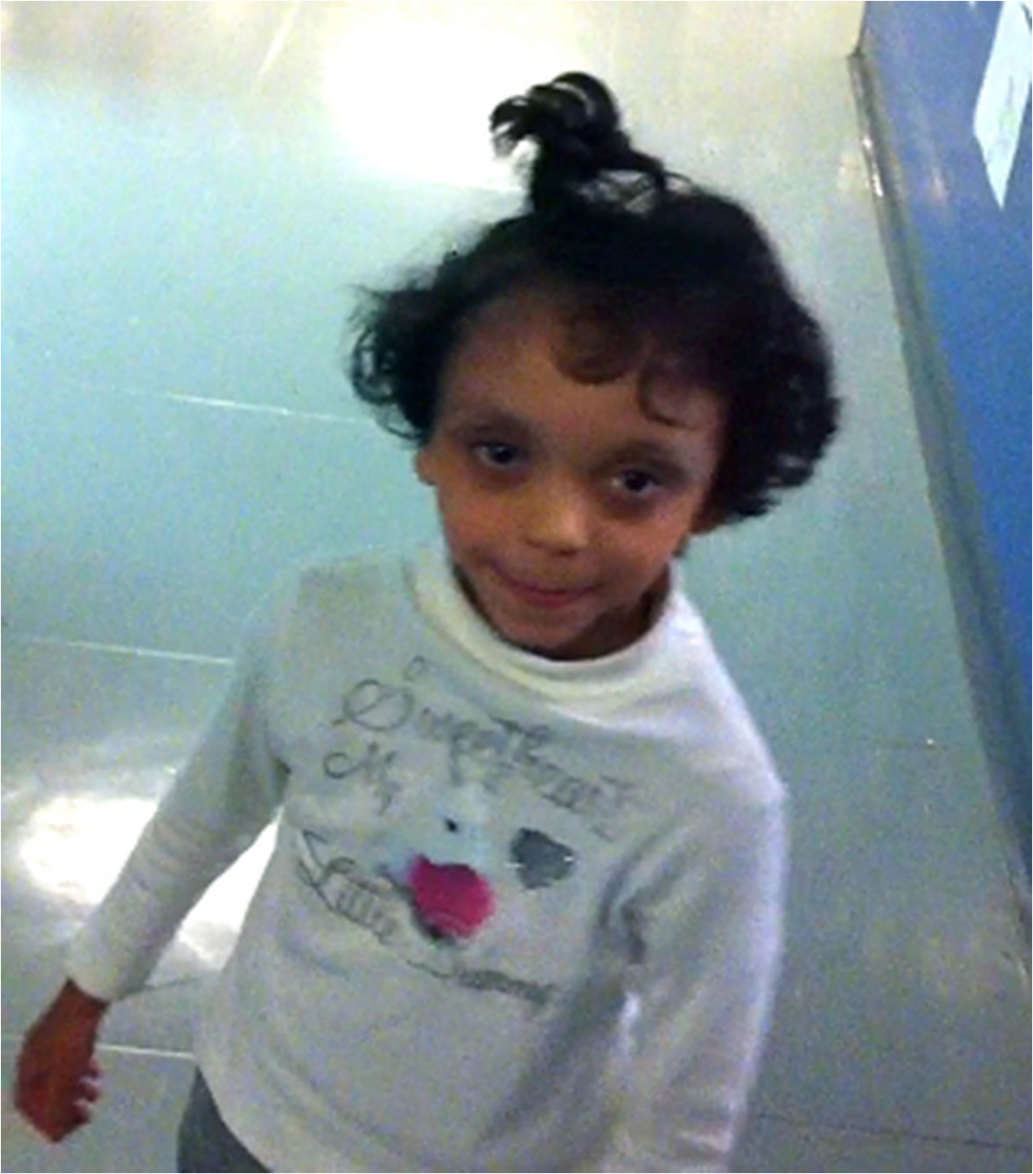
Facial features of the proband.

Additional physical features included: growth retardation, hypotonia, brachycephaly, midface hypoplasia, wide spaced front, hyperthelorisme, micrognatia, wide philtrum, depressed and wide nasal bridge, small teeth, deep-set eyes with downslanting palpebral fissures, normal ears (Figure 2).

Her developmental milestones were delayed as she walked at the age of 2 years and 6 month, her first words were spoken at the age of 6 years.

Heart ultrasound, electroencephalogram, liver-spleen ultrasound, visual and audiological examination were all normal.

Our research is in compliance with the Helsinki Declaration and written informed consent was obtained from the patient for the publication of this report and any accompanying images.

## Discussion

The 18q-syndrome is characterized by a deletion that involves the distal section of 18q and typically extends to the tip of the long arm. The breakpoint involved varies greatly and the presence or absence of specific clinical features may depend on the size and position of the deleted region [8]. The increasing use of molecular techniques such as array-CGH enables more accurate definition of the breakpoints. This, in turn, enables researchers to study which parts of the chromosome correlate with the different clinical features of the condition [9].

It is generally characterized by mental retardation, development delay, short stature, hearing loss, white matter abnormalities of the brain, neurologic abnormalities such hypoplasia, hands and feet malformation and ocular anomalies [5,6]. The facial dysmorphism may include brachycephaly, midface hypoplasia, everted lower lip, narrow upper lip with absent philtrum, downturned corners of the mouth, depressed and wide nasal bridge, small teeth, deep-set eyes with downslanting palpebral fissures, epicanthus, strabismus, prominent anthelix and antitragus, and very narrow external ear canal [10]. The associated anomalies include vertebral anomalies, genitourinary changes, congenital heart defects, cleft lip and palate [10].

It is thought that the decreased growth may be due to a growth hormone deficiency. This region contains the genes myelin basic protein (*MBP*) and the galanin receptor which are both candidates for the growth hormone insufficiency. The galanin receptor is involved in growth hormone response and is therefore a good candidate for the growth hormone insufficiency [9,11]. Interestingly, this region is almost identical to the region that has been identified to be responsible for the myelination problems. Thus, the gene or genes responsible for these two features are either the same gene or two tightly-linked genes. Up until now, all people who have been shown to have dysmyelination also have a growth hormone deficiency, so it has not yet been possible to uncouple these two features [9]. The common finding of narrow ear canals in 18q- has been linked, by three studies, to the loss of part of the region 18q22.3 [9,12].

A critical region for the microcephaly (small head) that is often present in 18q- has been shown to be located to band 18q21.33 [8,9].

Chromosome 1p duplications have been reported rarely and his contribute to patient’s phenotype it’s difficult to estimate. A review of the literature suggests that there is not possible to establish a syndrome. The most consistent manifestations are low birth weight, grow retardation, craniofacial and hand anomalies, and congenital heart disease. Most males had abnormal genitalia, whereas females had normal genitalia [7]. Apart from not specific features such as microcephaly, growth retardation, and developmental delay, the most striking feature reported in previous studies is metopic, sagittal and coronal synostosis [13].

Such opposing phenotypes, cranial synostosis in dup(1)(p36)and large late closing anterior fontanels in del(1)(p36) is probably corraleted to a gene that regulates suture closure at chromosome 1p36 [13]. Chromosome 1p36 rearrangements have been associated also with various neoplasms such as neuroblastoma suggesting that 1p36 region contain a tumor-suppressor gene involved in malignancy [13].

The coexistence of two chromosomal rearrangements complicates the clinical picture and creates a chimeric disorder marked by characteristics of both chromosomal anomalies.

The patient reported presents not specific characteristics like growth and psychomotor retardation and also feet malformation and microcephaly found in both anomalies.

The most clinical features as hypotonia, midface hypoplasia, micrognatia, wide philtrum, small teeth, deep-set eyes with downslanting palpebral fissures, umbilical hernia and RVU are related to 18q deletion syndrome.

The patient also present hyperthelorisme, depressed and wide nasal bridge probably related to 1p36.3 duplication.

Patient’s clinical phenotype isn’t completely superimposable to the one present in 18q syndrome, for example, hearing abnormality, one of the most consistent features with 66 per cent of children affected, it is absent in this patient. This is probably consequent to the great breakpoint variability and to the simultaneous presence of 1p duplication.

Concerning VUR for which the patient referred to our clinic, most researchers now acknowledge that VUR is genetically heterogeneous [2].

The initial evidence suggesting a genetic origin of primary VUR came from twin studies, showing an 80%–100% concordance for VUR in monozygotic twins vs. a 35-50% concordance in dizygotic twins [2].

Studies of humans with chromosomal abnormalities suggest for not syndromic primary VUR candidate loci or genes on chromosomes 6p, 10q26, 19q13 and 13q33-34 [2].

M.L. Conte et al showed best evidence of linkage with VUR on chromosome 3 p12.3-3q24 and on chromosome 1p36.32-1p34.3.

None of 13 reported patients affected by chromosome 1p36 terminal duplications evidenced the presence of VUR.

No part of the chromosome 18 has yet been identified related to VUR despite is reported approximately in 15% of patient affected by syndrome 18q.

## Conclusion

The clinical case discussed it is the first reported to be affected by both 18q-syndrome and 1p36 duplication (despite some children with 18q-syndrome have the involvement of an additional chromosome, usually a duplication of material from another chromosome).

The coexistence of two chromosomal rearrangements complicates the clinical picture and creates a chimeric disorder marked by characteristics of both chromosomal anomalies.

Chromosome 1p duplications is a rare anomaly with only 13 cases reports in literature his contribute to patient’s phenotype it’s difficult to estimate [6].

Concerning VUR that interest the syndromic disease presented, we know that none of 13 reported patients affected by chromosome 1p36 terminal duplications evidenced the presence of VUR and no part of the chromosome 18 has yet been identified related to VUR despite is reported approximately in 15% of patient affected by syndrome 18q.

Most researcher now acknowledge that VUR is genetically heterogeneous and no more these loci or genes has been shown causally related to primary VUR.

Continuous increasing knowledge of the genetic basis of VUR should help us to further understand its pathogenesis.

## Consent

“Written informed consent was obtained from the patient’s guardian/parent/next in keen for publication of this report and any accompanying images”.

## Competing interests

The authors declare that they have no competing interests.

## Authors’ contributions

All authors read and approved the final manuscript.
